# HIV-Positive Status Disclosure and Associated Factors among Children in North Gondar, Northwest Ethiopia

**DOI:** 10.5402/2012/485720

**Published:** 2012-12-13

**Authors:** Digsu Negese, Kefyalew Addis, Akilew Awoke, Zelalem Birhanu, Dagnachew Muluye, Sisay Yifru, Berihun Megabiaw

**Affiliations:** ^1^Institute of Public Health, College of Medicine and Health Sciences, University of Gondar, P.O. BOX 196, Gondar, Ethiopia; ^2^School of Biomedical and Laboratory Sciences, College of Medicine and Health Sciences, University of Gondar, P.O. BOX 196, Gondar, Ethiopia; ^3^School of Medicine, College of Medicine and Health Sciences, University of Gondar, P.O. BOX 196, Gondar, Ethiopia

## Abstract

*Introduction*. Clinical reports have indicated positive outcomes associated with disclosure of HIV-positive status in children. This study assessed the level and associated factors of HIV-positive status disclosure to HIV-infected children in northwest Ethiopia. *Methods*. Institution-based cross-sectional study was conducted among HIV-positive children from March to April 2012. Data were collected using a structured questionnaire by face-to-face interview technique. Bivariate and multivariate analyses were performed. *Results*. Of the 428 children, 169 (39.5%) were disclosed their HIV-positive status. The mean age of HIV-positive status disclosure was at 10.7 (±2.3) years. Having a nonbiological parent (AOR = 4.14, 95% CI: 1.22, 14.04), child's age older than 10 years (AOR = 8.54, 95% CI: 4.5, 15.53), and death of a family member (AOR = 2.04, 95% CI: 1.16, 3.6) were significantly and independently associated with disclosure of HIV-positive status to infected children. *Conclusions*. The rate of disclosure of HIV-positive status to infected children still remains low in North Gondar. Hence, it is important to target children living with their biological parents and having young parents and children younger than 10 years. The guideline for disclosure of children with HIV/AIDS should be established in an Ethiopian context.

## 1. Introduction 

HIV/AIDS is increasingly affecting the health and welfare of children and undermining hard-won gains of child survival in highly affected countries [1]. Recent estimates from the Joint United Nations Programs on HIV/AIDS (UNAIDS) suggest that globally about 2.5 million children younger than 15 years of age are infected with HIV: 90% living in sub-Saharan Africa [2] and about 64,813 living in Ethiopia [3]. Without treatment 75% of HIV-infected children will die before their fifth birthday [4]. As highly active antiretroviral therapy (HAART) becomes increasingly available in low resource settings, infected children are living longer [5]. With increased survival, one of the greatest psychosocial challenges that parents and caregivers of HIV-infected children face is the disclosure of HIV-positive status to their infected children.

One of the most difficult issues that families with HIV-infected children face is when and how to talk about HIV to their children. HIV-positive status disclosure to infected children and adolescents should take place in a supportive environment with collaboration and cooperation among caregivers and health care providers. Disclosure is contingent on the caregiver's acknowledgement of the illness, the readiness to disclose, and child's cognitive skills and emotional maturity [6]. 

Despite emerging evidence of the benefit of disclosure, when and how to disclose the diagnosis of HIV to children remain a clinical dilemma [7]. Clinicians and other members of multidisciplinary teams should collaborate with caregivers of HIV-infected children to disclose HIV diagnosis to the child in a developmentally appropriate manner [6].

Children react to HIV disclosure in different ways and it is not uncommon for relatives to disagree about disclosing HIV-related information to children. Disclosure has to be individualized taking into consideration the particular child, parent (s), family, household, and community. HIV diagnosis disclosure entails communication about a potentially life-threatening, stigmatized, and transmissible illness, and many caregivers fear that such communications may create distress for the child [5]. 

The American Academy of Pediatrics strongly encourages disclosure of HIV-positive status to school-age children [8]. But in Ethiopia, no such recommendations and guidelines are available concerning disclosure of pediatric HIV, and disclosing the diagnosis of HIV or AIDS to a child is controversial and challenging among health care providers, parents, and caregivers. Thus this study assessed the magnitude of HIV-positive status disclosure and the associated factors among HIV-infected children in Northwest Ethiopia.

## 2. Methods 

### 2.1. Study Design, Period, and Setting

An institution-based cross sectional study design was carried out from March to April, 2012 at the three hospitals of North Gondar Zone. North Gondar Zone is one of the 11 zones in the Amhara National Regional State.

### 2.2. Study Population and Sampling Procedures

All HIV-positive children aged 5–15 years who were on care and support followup at the pediatric ART clinics of the three hospitals (Gondar, Metema, and Dabark) in North Gondar Zone. All caregivers of the children enrolled in the chronic HIV care at pediatric ART units of the three hospitals were included. Children who came by themselves or with no caregiver or parent were excluded because of ethical concerns.

### 2.3. Definitions

Disclosure refers to when the caregiver said that the child knows his/her HIV/AIDS diagnosis regardless of who told the child.

### 2.4. Data Collection and Management

Data were collected by an interview technique using a structured questionnaire which was first prepared in English then translated to the local language Amharic. A clinical nurse working at the pediatric ART clinic of each hospital and supervised by a supervisor collected the data. The prepared questionnaire was pretested and structured accordingly in a logical manner into sociodemographic, clinical characteristics and HIV-positive disclosure parts. The returned questionnaires were checked for completeness on site by the supervisor. The data were entered in to EPI INFO version 3.5.1 statistical software and analyzed by SPSS version 20.0. Frequencies and cross-tabulations were used to summarize descriptive statistics. Bivariate and multivariate analyses were performed to test associations. Variables having *P* value ≤ 0.2 in the bivariate analysis were entered into a multiple logistic regression model to control the confounding effect. Odds ratios with their 95% confidence intervals were calculated to measure associations, and statistical significance was set at *P* < 0.05. Efforts were made to assess whether the necessary assumptions for the application of multiple logistic regression were fulfilled. In this regard, the Hosmer and Lemeshow goodness-of-fit test was considered, and *P* value > 0.05 is considered as a good fit model.

### 2.5. Ethical Considerations

Ethical clearance was obtained from the Ethical Review Board of the University of Gondar. Permission was obtained from the hospitals administration and the ART focal persons at each hospital. After the purpose of the study was explained, verbal consent was obtained from each caregiver. Interviews were carried out privately in a separate room in the hospitals. Participants also were informed that participation was on voluntary basis and that they can withdraw at any time if they are not comfortable about the questionnaire. Names or personal identifiers were not included in the written questionnaires to ensure participants' confidentiality.

## 3. Results

### 3.1. Socio-Demographic Characteristics

A total of 428 caregivers were interviewed. Of these, 343 (80.1%) were from Gondar university referral hospital. Three hundred thirty-one (77.3%) of the caregivers were females, 368 (86%) were orthodox Christians, and the majority (89.5%) were urban residents. About half (51.4%) of the caregivers had a monthly income of 300–999 Ethiopian Birr per month. Nearly two thirds (65.4%) of the caregivers were biological parents of the children and one third were daily labourers. 

Almost half (49.3%) of children were males and the mean age of children was 9.96 ± 3.0 SD years. The median age at diagnosis of HIV was 6.0 years (IQR = 5 years). Three hundred four (71%) of the children attended their primary school and nearly two third of them were living with their biological parents (Table 1).

### 3.2. Clinical Characteristics

Nearly two third (61.9%) of the caregivers were HIV-positive of whom 92.5% were on ART and 86.4% had disclosed their HIV-positive status to someone else. 

Majority (81.3%) of the children had a WHO clinical stage I disease. Majority, that is, 344 (80.4%) children, had history of opportunistic infections (OIs) and 42.5% were hospitalized. Three hundred forty-eight (81.3%) children were on ART at the date of interview (Table 2).

### 3.3. Magnitude of HIV-Positive Status Disclosure

**Figure 1:**
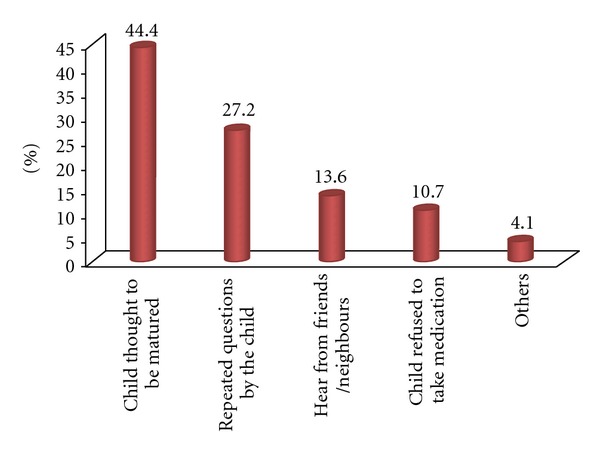
Reasons for disclosing HIV-positive status to HIV-positive children in North Gondar Zone, Northwest Ethiopia, 2012.

**Figure 2:**
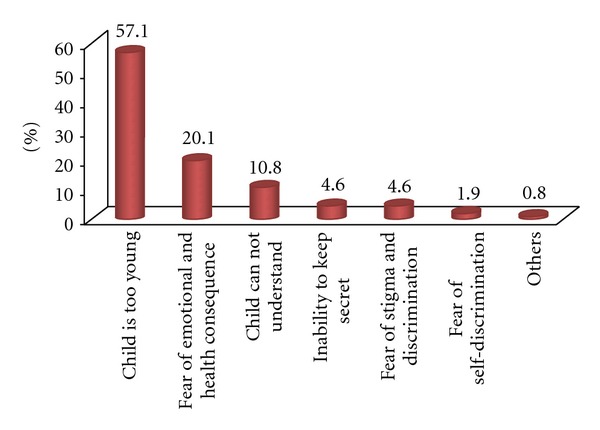
Reasons for not disclosing HIV-positive status to HIV-positive children in North Gondar Zone, Northwest Ethiopia, 2012.

Of the 428 children, 169 (39.5%, 95% CI: 34.8, 43.7) of the children living with HIV/AIDS were disclosed their HIV-positive status. The mean age at disclosure was 10.7 years (±2.3 years). Sixty-nine (40.8%) children were disclosed by their biological parents while 38.5% of children were disclosed by health care providers. Sixty-seven (39.6%) of the disclosers were HIV-positive. The prominent reasons for disclosure as mentioned by caregivers were “child thought to be matured” (44.4%) and repeated questionings of “what happened to me” (27.2%) by the child (Figure 1). Participants mentioned reasons for not disclosing the child about his/her HIV-positive status. More than half still believe that the child is too young (57.1%) and another one fifth fear the negative emotional and health consequence (20.1%) of disclosure (Figure 2). Two hundred twenty-one (81.1%) of the caregivers believed that disclosing the HIV-positive status to the child is advantageous and three quarters (76.8%) had the intension to disclose in the near future.

### 3.4. Factors Associated with HIV-Positive Status Disclosure

As clearly depicted on the multivariate logistic regression, caregiver's relation with the child, age of the child and loss of a family member were independently and significantly associated with disclosure of HIV-positive status to HIV-infected children. However, factors related to the caregiver such as sex, religion, HIV-positive status, and educational status, as well as sex of the child, history of OIs, and ART status of children were not significantly associated with disclosure of HIV-positive status to HIV-infected children. 

Accordingly, nonbiological parents were 4.14 (AOR = 4.14, 95% CI: 1.22, 14.04) times more likely to disclose HIV-positive status to HIV-infected children as compared to biological ones. Age of the child was one of the factors significantly associated with disclosure of HIV-positive status in which children older than 10 years of age were 8.54 (AOR = 8.54, 95% CI: 4.5, 15.53) times more likely to be disclosed as compared their counterparts. Those children who lost any of their family members were two (AOR = 2.04, 95% CI: 1.16, 3.6) times more likely to be disclosed their HIV-positive status as compared to their counterparts (Table 3).

## 4. Discussion 

In Ethiopia, due to the recent improvements in access to antiretroviral therapy, dramatic decline of mortality and morbidity of HIV-infected children has been observed [9]. As children survived for longer periods of time, disclosure issues emerge related to pubertal development and sexuality, fear of transmission, and the need to promote adherence to complex and often toxic regimens [10]. Studies have indicated positive outcomes associated with HIV-positive status disclosure. Promotion of trust, improved adherence, open family communication, and better long-term health and emotional well-being in children are some of the advantages [8].

 In this study, 39.5% of HIV-positive children were disclosed their HIV-positive status. This finding is similar to studies conducted in USA which reported a disclosure rate of 35–43% [11–13]. But it is very low as compared to studies done in high-income countries in which the disclosure rate ranges from 57 to 100% [10, 14, 15]. The lower prevalence of disclosure in our study might be due to fear of stigma and discrimination by the family members. Since the majority of HIV-infected children acquired the virus from their mothers, disclosure of a child's HIV-positive diagnosis often leads to disclosure of other family secrets that leads to stigma and discrimination. Caregiver's perceived lack of emotional preparedness of children and [16, 17] and the absence of recommendations and guidelines for disclosure of HIV-positive children in Ethiopia might have also contributed for the lower rate of disclosure [8].

This finding was somewhat higher as compared to studies conducted in Poland (16.2%) [18], Thailand (30.1%) [19], Ghana (21%) [7], and Nigeria (13.5%) [20]. It is also higher as compared to a study conducted in Addis Ababa, Ethiopia (17.4%) [16]. The possible justification can be difference in time period and there might be also increased awareness on the benefit of disclosure by caregivers. Additionally, this study assessed disclosure status among children 5–15 years of age, but the study conducted in Addis Ababa includes all pediatric age groups. 

Age was identified as a factor for disclosure in this study and in another study conducted in Ethiopia [16]. This could be due to the caregivers' belief that at early age, the child is lacking the emotional and cognitive maturity needed to understand the disease and its implications [12, 13, 21, 22]. In this study, the mean age at disclosure was 10.7 years which was high as compared to studies done in New York (7 years) and Nigeria (8.7 years) but somewhat comparable with a study conducted in Ghana (11.72 years) [7, 17, 20]. Reasons cited by the caregivers were consistent with that of studies in resource-limited countries; namely, child is too young, fear of emotional and health consequences, fear of stigma and discrimination, and fear that the child would not keep diagnosis to themselves. Caregivers believed their children were too young to know their status [7]. 

In our study, the factors that were independently and significantly associated with disclosure were the age of the child, nonbiological parent relation with the child, and loss of family member. Consistent with previous studies done in Ghana and London, children were more likely to be disclosed if they were orphaned [11, 23].

The results of our study supported previous studies done in Nigeria, Thailand, London, and Massachusetts [11, 19, 20, 23] that showed older age of infected children as a determinant factor for HIV-positive status disclosure. Children older than 10 years were more likely to be disclosed than those younger than 10 years. The child's theory of cognitive understanding of illness is also in favour of this finding. Accordingly, the age from 9 to 10 years and older is considered to be the best time for HIV-infected children to know about their sickness as at this age children can understand about the complex causes of illness and its consequences [19]. 

In this study, nonbiological caregivers were more likely to disclose the child's HIV-positive status than biological caregivers. This finding is in agreement with studies done in Philadelphia and Thailand [19, 21] where most children who knew their diagnosis were living with caregivers who were not related to them, whereas the majority of children who did not know the diagnosis were living with biological parents. As argued by these studies biological parents might not be willing to confront the fact of their own responsibilities in passing the infection onto their children. 

This study has the following strengths and limitations. The sample size is relatively larger than other studies done in sub-Saharan Africa, and generalization can be made to children on chronic HIV/AIDS care in Ethiopia. But as a cross-sectional study, the associations observed may not be causal. Because of lack of data on adherence to treatment, we could not include it in the analysis. Furthermore, the study did not explore the benefits of disclosure on adherence and clinical improvement in HIV/AIDS.

## 5. Conclusions

The rate of disclosure of HIV-positive status to HIV-infected children is low in this study. Non biological parent caregivers, children older than 10 years of age, and loss of family member were independently and significantly associated with disclosure of HIV-positive status to HIV-infected children. Hence, it is important to target young children living with their biological parents and those having young parents. Guideline for disclosure of children with HIV/AIDS has to be established in Ethiopian context. We recommend further studies to be undertaken to explore the benefits of disclosure of HIV-positive status to HIV-infected children. 

## Figures and Tables

**Table 1:** Sociodemographic characteristics of caregivers and children in North Gondar Zone, Northwest Ethiopia, 2012 (*n* = 428).

Variables	Frequency	Percent
Site of data collection		
Gondar university hospital	343	80.1
Dabark hospital	57	13.3
Metema hospital	28	6.5
Sex of caregiver		
Male	97	22.7
Female	331	77.3
Age		
≤30	126	29.4
31–40	173	40.4
41–50	64	15.0
51–60	32	7.5
≥61	33	7.7
Religion of caregiver		
Orthodox christian	368	86.0
Muslim	43	10.0
Protestant	17	3.9
Residence of the caregiver		
Urban	383	89.5
Rural	45	10.5
Monthly family income in Birr		
<300	93	21.7
300–999	220	51.4
≥1000	115	26.9
Relation with the child		
Biological parent	280	65.4
Grandparent	63	14.7
Siblings	29	6.8
Relatives	41	9.6
Others	15	3.5
Educational status of the caregiver		
No formal education	168	39.2
Primary school (1–8)	114	26.6
Secondary school (9–12)	115	26.9
Above secondary school	31	7.2
Occupation of caregiver		
House wife	114	26.6
Government employed	57	13.3
Farmer	23	5.4
Merchant	61	14.3
Daily labourer	144	33.6
Others	29	6.8
Sex of child		
Male	211	49.3
Female	217	50.7
Age of child		
<10	203	47.4
≥10	225	52.6
Educational status of child		
Not started education	61	14.3
Kindergarten	48	11.2
Primary school (1–8)	304	71
Secondary school (9–12)	15	3.5
With whom currently living		
Biological parent	284	66.4
Siblings	27	6.3
Relatives	100	23.4
At orphanage camp	12	2.8
Others	5	1.2
Lost any of his/her families		
Yes	237	55.4
No	191	44.6
Lost who *n* = 237		
Mother only	58	24.5
Father only	85	35.8
Both mother and father	94	39.7

**Table 2:** Clinical characteristics of caregivers and children in North Gondar Zone, Northwest Ethiopia, 2012.

Variables	Frequency	Percent
HIV-positive status of the caregiver		
Positive	265	61.9
Negative	112	26.2
Not tested	51	11.9
ART status of caregiver, *n* = 265		
On ART	245	92.5
Before ART	20	7.5
Disclosure of HIV-positive status of the caregiver, *n* = 265		
Yes	229	86.4
No	36	13.6
WHO clinical staging		
I	348	81.3
II	42	9.8
III	33	7.7
IV	5	1.2
History of OIs		
Yes	344	80.4
No	84	19.6
History of hospitalization		
Yes	182	42.5
No	246	57.5
ART status of child		
On ART	348	81.3
Before ART	80	18.7

**Table 3:** Bivariate and multivariate analysis of factors associated with disclosure of HIV-positive status to HIV-infected children in North Gondar Zone, Northwest Ethiopia, 2012.

Variables	Disclosure status		Crude OR (95% CI)	Adjusted OR (95% CI)
	Disclosed	Not disclosed		
Sex of caregiver				
Male	34	63	0.78 (0.49, 1.25)	
Female	135	196	1.00	
Age of caregiver				
≤30	33	93	1.00	
31–40	66	107	1.74 (1.05, 2.87)	
41–50	28	36	2.19 (1.16, 4.13)	
51–60	16	16	2.89 (1.27, 6.26)	
>60	26	7	10.47 (4.15, 26.38)	
Religion of caregiver				
Orthodox christian	144	224	1.00	
Muslim	13	30	0.67 (0.34, 1.34)	
Protestant	12	5	3.73 (1.29, 10.82)	
Relation with the child				
Biological parent	83	197	1.00	1.00
Not biological parent	86	62	3.29 (2.17, 4.99)	**4.14 (1.22, 14.04)**
Educational status of caregivers				
No formal education	72	97	1.56 (0.69, 3.51)	
Primary school	36	77	0.98 (0.42, 2.3)	
Secondary school	51	64	1.67 (0.72, 3.87)	
Above secondary school	10	21	1.00	
Sex of child				
Male	88	123	1.20 (0.81, 1.77)	
Female	81	136	1.00	
Age of child				
<10	26	177	1.00	1.00
≥10	143	82	11.87 (7.25, 19.44)	**8.54 (4.5, 15.53)**
Educational status of child				
Not started education	7	54	1.00	
Kindergarten	2	46	0.335 (.07, 1.69)	
Primary school (1–8)	150	154	7.51 (3.31, 17.04)	
Secondary school (9–12)	10	5	15.43 (4.07, 58.41)	
With whom currently living				
Biological parent	88	196	1.00	
Siblings	13	14	2.07 (93, 4.58)	
Relatives	54	46	2.62 (1.64, 4.17)	
At orphanage camp and others	14	3	10.39 (2.91, 37.09)	
HIV-positive status of caregivers				
Positive	78	187	1.00	
Negative	60	52	2.76 (1.75, 4.36)	
Unknown status	31	20	3.72 (1.99, 6.92)	
Lost any of his/her family				
Yes	119	118	2.84 (1.89, 4.29)	**2.04 (1.16, 3.6)**
No	50	141	1.00	1.00
History of OIs				
Yes	147	197	2.10 (1.23, 3.57 )	
No	22	62	1.00	
ART status of the child				
On ART	146	202	1.79 (1.05, 3.04)	
Before ART	23	57	1.00	
